# Persistence of *Brucella abortus* in the Bone Marrow of Infected Mice

**DOI:** 10.1155/2018/5370414

**Published:** 2018-12-03

**Authors:** Cristina Gutiérrez-Jiménez, Lisiena Hysenaj, Alejandro Alfaro-Alarcón, Ricardo Mora-Cartín, Vilma Arce-Gorvel, Edgardo Moreno, Jean Pierre Gorvel, Elías Barquero-Calvo

**Affiliations:** ^1^Programa de Investigación en Enfermedades Tropicales, Escuela de Medicina Veterinaria, Universidad Nacional, Heredia, Costa Rica; ^2^Aix Marseille University, CNRS, INSERM, CIML, Marseille, France; ^3^Pathology Department, Escuela de Medicina Veterinaria, Universidad Nacional, Heredia, Costa Rica

## Abstract

Brucellosis is a zoonotic bacterial infection that may persist for long periods causing relapses in antibiotic-treated patients. The ability of *Brucella* to develop chronic infections is linked to their capacity to invade and replicate within the mononuclear phagocyte system, including the bone marrow (BM). Persistence of *Brucella* in the BM has been associated with hematological complications such as neutropenia, thrombocytopenia, anemia, and pancytopenia in human patients. In the mouse model, we observed that the number of *Brucella abortus* in the BM remained constant for up to 168 days of postinfection. This persistence was associated with histopathological changes, accompanied by augmented numbers of BM myeloid GMP progenitors, PMNs, and CD4^+^ lymphocytes during the acute phase (eight days) of the infection in the BM. Monocytes, PMNs, and GMP cells were identified as the cells harboring *Brucella* in the BM. We propose that the BM is an essential niche for the bacterium to establish long-lasting infections and that infected PMNs may serve as vehicles for dispersion of *Brucella* organisms, following the Trojan horse hypothesis. Monocytes are solid candidates for *Brucella* reservoirs in the BM.

## 1. Introduction

Brucellosis is a zoonotic bacterial infection caused by members of the genus *Brucella* [1]. In humans, the disease is long-lasting, displaying a variety of clinical and pathological manifestations that may persist for months or years [2–5]. If the infection is not properly treated, it may cause death.

The ability of *Brucella* organisms to develop chronic infections is linked to their competence to invade the mononuclear phagocyte system, where they replicate within the endoplasmic reticulum [6]. In addition, the poor proinflammatory responses induced at the onset of the infection [7], together with the capacity of *Brucella* organisms to extend the life of infected cells, are factors that contribute to the pathogenicity of this microorganism [7, 8].

The persistence of *Brucella* organisms in humans occurs in the lymph nodes, spleen, liver, bone marrow (BM), reproductive organs, and joints [9, 10]. The bacterium is isolated from the BM in about half of the human patients with brucellosis [4]. However, in all brucellosis cases, the BM displays histopathological alterations, whether or not the bacterium is isolated from this tissue. Common hematological signs are neutropenia, thrombocytopenia, and anemia, and in severe cases, pancytopenia has also been reported [4, 5, 11]. In most patients, the BM cellular alterations ameliorate or disappear after antibiotic treatment [4]. Moreover, brucellosis transmission by BM transplantation from seemingly healthy donors has been reported [12]. These data indicate that even in those cases in which the bacterium is not isolated from the BM, it still may be present, hidden within cells.

Following experimentation in mice, it has been proposed the BM may be the most relevant tissue for *Brucella* persistence [13]. In addition, *Brucella canis* has been shown to persevere in the BM at chronic stages of mouse infection [14]. Here, we describe the persistence of *Brucella abortus* in cells of the mice BM and propose that this tissue is essential for establishing long-lasting chronic infections.

## 2. Materials and Methods

### 2.1. Infection Protocols

*B. abortus* 2308W expressing red fluorescent protein from *Discosoma* coral (*B. abortus*-RFP), provided by Jean-Jacques Letesson (University Notre-Dame de la Paix, Namur, Belgium) was used in all experiments. BALB/c mice were supplied by the Escuela de Medicina Veterinaria, Universidad Nacional, Costa Rica, and Laboratorio de Ensayos Biológicos, Universidad de Costa Rica. C57BL/6 mice were purchased from Charles River Laboratories (Les Oncins, France), housed under specific pathogen-free conditions, and handled in accordance with French and European guidelines.

Mice were infected by the intraperitoneal route (i.p.) with 10^6^ bacterial colony forming units (CFU) of *B. abortus*-RFP. At different phases of the infection, the spleen, liver, lymph nodes, and bone marrow (BM) were collected. Then, the organs subjected to bacterial counts, histopathological examination, and cells analyzed by flow cytometry, as described elsewhere [15, 16]. Experimentation in mice was conducted following the guidelines and consent of the “Comité Institucional para el Cuido y Uso de los Animales de la Universidad de Costa Rica” (CICUA-47-12) and in accordance with the corresponding Animal Welfare Law of Costa Rica (Law 9458). All animals were kept in cages with food and water *ad libitum* under biosafety containment conditions.

BM cells were also isolated and infected ex vivo in the presence of anti-*Brucella* antibodies, following previous protocols [16]. Briefly, BM cells were isolated from the tibia and femur of *B. abortus*-RFP-infected mice at 8 and 30 days of postinfection by flushing bones with HBSS (no calcium, no magnesium) or RPMI medium. BM cells were then incubated with *B. abortus*-RFP at MOI of 50 bacteria/cell at 37°C for 2 hours, washed with PBS, suspended in HBSS, and subjected to examination. The number of CFUs infecting enriched BM-derived PMNs was estimated by lysing the cells and counting bacteria in agar plates [17].

### 2.2. Immunofluorescence

BM cells (50 to 100 *μ*l resuspended in DMEM at a concentration of 10^6^ cells/ml) were loaded on alcian blue-coated coverslips (Sigma) and incubated for 20 min at 37°C to allow cell attachment. Twenty minutes Antigenfix (Diapath) was used for fixation. Once fixed onto coverslips, cells were washed with PBS and slides were mounted using ProLong Gold Antifade reagent containing DAPI (Thermo Fisher Scientific). Slides were observed with confocal microscope (Leica TCS SP8) as described before [18]. Image analyses were performed using the ZEN 2011 software.

### 2.3. Histopathology

For histopathological studies, the spleen, lymph nodes, and BM from infected and PBS-treated mice were fixed in 10% neutral buffered formalin, processed and stained with hematoxylin and eosin or Giemsa stain [19]. The histopathological score (from 0 (negative) to 4 (severe)) was determined by semiquantitative analysis as previously described [20–22].

### 2.4. Flow Cytometry

For flow cytometric analyses, cell surface markers were stained using the following antibodies: BV421 anti-CD11b (M170), BV711 anti-Ly6G (1A8), BV785 anti-F4/80 (BM8), and BV570 anti-CD4 (RM4–5) antibodies were purchased from BioLegend; AF647 anti-CD34 (RAM34), BV711 anti-CD8*α* (53-6.7), and BV650 anti-CD3 (245-2CII) from BD Biosciences; and Alexa Fluor 488 and APC both anti-CD115 (AFS98), PE anti- Ly6G (1A8), Ef450 anti-CD45R/B220 (RA3-6B2), PE Cy7 anti-CD19 (1D3), and AF700 anti-CD44 (1M7) antibodies from eBiosciences; and APC Cy7 anti-CD16/32 (2.4G2), BV510 anti-Sca-1 (D7), and BV605 CD117/c-kit (2B8) from BD Biosciences. An antibody staining scheme is provided in Table S1. Cells were identified according to the staining scheme and the percentage of each cell type determined in relation to all living cells of bone marrow at 8 and 30 days of postinfection. Cell viability was evaluated using Fixable Viability Dye UV (eBiosciences). Cells were fixed with Antigenfix for 20 min before the acquisition. Multiparameter flow cytometry was performed using a FACS LSRII UV (BD Biosciences) or Guava easyCyte (Millipore). Flow cytometry data were analyzed using the FlowJo software, version 10.0.7 (Tree Star Inc.).

### 2.5. Statistics

One-way analysis of variance (ANOVA) followed by Dunnett's test or multivariate analysis of variance (MANOVA) was used to determine statistical significance in the different assays. The JMP (https://www.jmp.com) and GraphPad Prism software (https://www.graphpad.com) were used for statistical analysis. Data were processed in Microsoft Office Excel 2015.

## 3. Results

**Figure 1:**
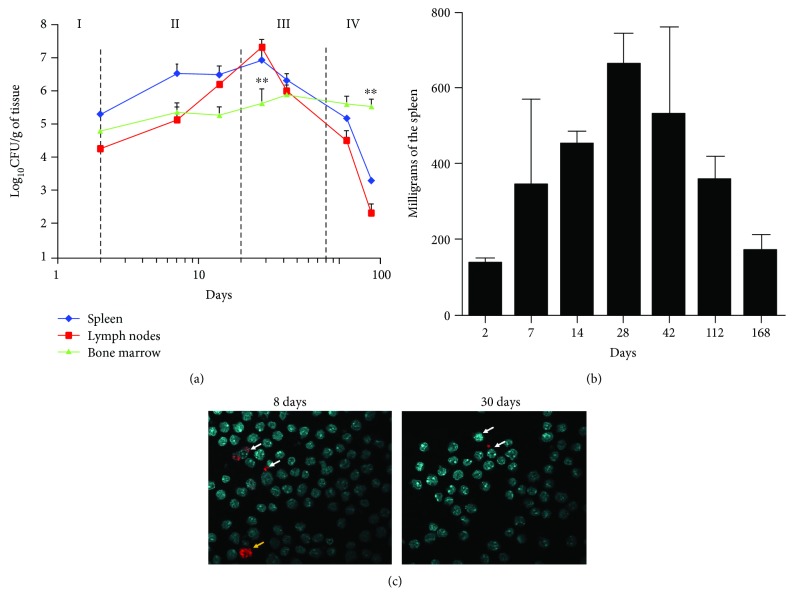
*B. abortus* persists in bone marrow during the course of infection. Mice were infected with *B. abortus*-RFP. (a) Spleen, lymph nodes, and BM were collected, and CFUs determined at different phases of infection [23]: the onset of infection (I), the acute phase (II), the chronic steady phase (III), and the chronic declining phase (IV). Each bar is the mean (±1 SD) of an experiment. Values of ^∗∗^ *p* < 0.01 are indicated in relation to spleen and lymph node bacterial loads. (b) Before CFU determination, the spleens were weighted at each time of examination. (c) BM cells were isolated from the tibia and femur of *B. abortus-*RFP- (red intracellular bacteria) infected mice at 8 and 30 days of postinfection mounted using ProLong Gold containing DAPI (blue nuclei). Microscope images are captured at 60x magnification confocal microscope.

According to bacterial loads, histopathological alterations, and immune response, murine brucellosis has been divided into four stages: onset of infection, acute phase, chronic phase, and chronic declining phase [23] (Figure 1(a)). After infection, *B. abortus* CFU counting was performed from the spleen, lymph nodes, and BM during the lapse of 168 days of postinfection (Figure 1(a)). Bacterial loads and kinetic profiles of the spleen and lymph nodes were similar. A significant bacterial increase was observed in the lymph nodes and spleen at the chronic steady phase III (28 days of postinfection), followed by a decrease in the bacterial numbers at the chronic declining phase IV and until the end of the experimentation. In the BM, *B. abortus* infection steadily persisted throughout all four phases, until day 168, when the CFU/g loads were significantly higher than those of lymph nodes and spleen. Similar results were obtained with C57BL/6 mice (not shown). The weight of the spleens increased until day 28 and then decreased until the end of the experiment, following a pattern similar to that of the kinetics of the CFU count (Figure 1(b)). Even though the number of CFU/g of BM was relatively high, the absolute numbers of *B. abortus* BM-infected cells were low at 8 (acute phase) and 30 (chronic phase) days of postinfection, suggesting that few infected cells harbored many bacteria (Figure 1(c)). However, a high number of bacteria was observed in some cells, a phenomenon that may account for the discrepancy between the CFU/g and the number of infected cells.

**Figure 2:**
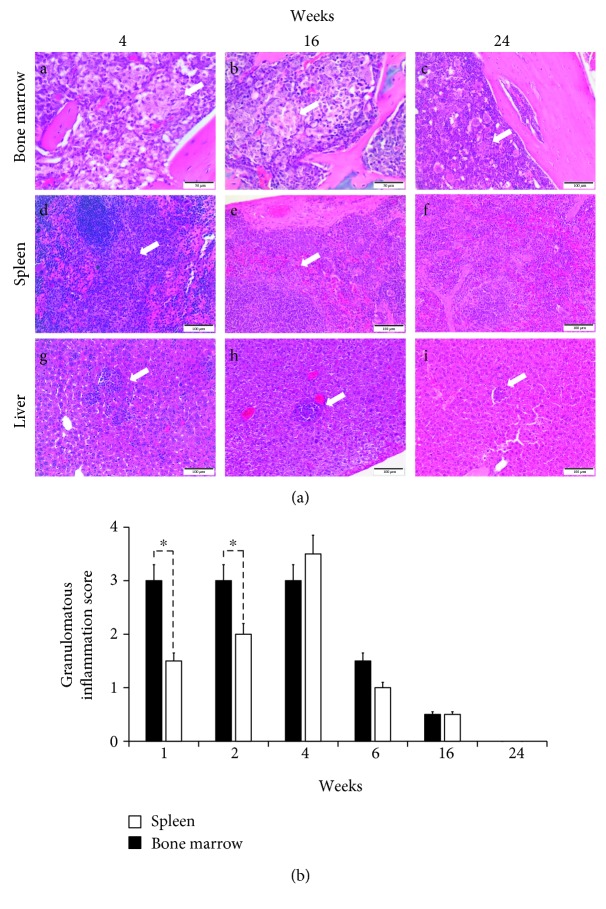
*Brucella abortus* induces a granulomatous inflammation in bone marrow. (a) Mice were infected with *B. abortus*-RFP. The spleen, liver, and BM were collected at different phases of infection and subjected to histopathological examination. (b) Granulomatous inflammation was scored from 0 (negative) to 4 (severe) [19] in BM over time. Each bar is the mean (±1 SD) of an experiment. Value of ^∗^ *p* < 0.05 is indicated in relation to BM and spleen granulomatous inflammation.

It has been demonstrated that most *Brucella*-infected human patients display histopathological alterations, whether or not the bacterium is isolated from the BM [4]. As shown in Figure 2(a), granulomatous inflammation was more severe and diffuse at acute stages than the multifocal chronic phase in the BM, spleen, and liver. At the acute phase, the inflammatory process was characterized by coalescing to diffuse inflammation with larger and cell-rich granulomas, while in the chronic phase, granulomatous inflammation was multifocal with smaller and fewer cellular lymphohistiocytic aggregates. Epithelioid macrophages predominate during the inflammatory process at early stages, reducing in number with chronicity. Classical granuloma formation was observed more clearly in the spleen and liver, while bone marrow developed an epithelioid macrophage-rich aggregate with scattered lymphocytes, which reduced its size and cellularity over time. Compared to the spleen, bone marrow granulomatous inflammation was more severe in the first two weeks of infection. After four weeks of infection, the spleen and bone marrow presented similar inflammation scores, though the granulomatous inflammation decreased in both tissues afterward (Figure 2(b)).

**Figure 3:**
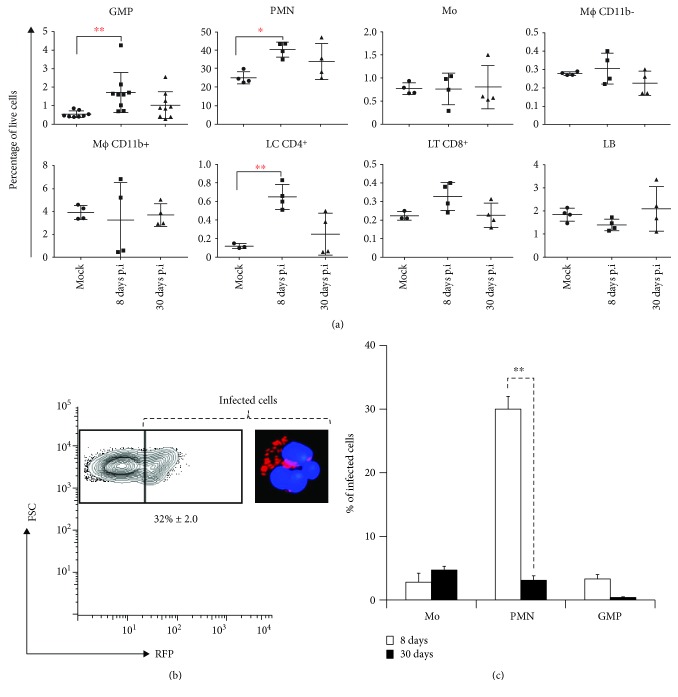
Bone marrow leukocyte variation at different stages of infection. (a) BM cells from *B. abortus*-infected mice were collected and subjected to multiparameter flow cytometry analysis. Cells were identified according to the staining scheme (Table S1) and the percentage of each cell type determined in relation to all living cells of bone marrow at 8 and 30 days of postinfection. Values of ^∗^ *p* < 0.05 or ^∗∗^ *p* < 0.01 are indicated in relation to control noninfected mice at 8 and 30 days of postinfection. (b) Whole BM cells were collected and infected ex vivo with *B. abortus*-RFP. Infected cells were gated based on the RFP (red) positivity, and the total percentages of infected cells were quantified. (c) BM cells from *B. abortus*-RFP-infected mice were collected and subjected to multiparameter flow cytometry analysis. Infected cells were gated based on the RFP (red) positivity, identified and quantified according to the staining scheme (Table S1) at 8 a 30 days of postinfection. Each bar is the mean (±1 SD) of an experiment. Values of ^∗∗^ *p* < 0.01 are indicated.

The cellular changes in the BM of infected mice were estimated by flow cytometry. At 8 days of postinfection, we observed changes in the hematopoietic cell population. At day 8 of postinfection, the percentage megakaryocyte-erythrocyte progenitor (MEP) decreased compared to the BM of noninfected mice (data not shown). Contrarily, the percentage of granulocyte-monocyte progenitors (GMP) significantly increased. Likewise, neutrophils (PMN) and CD4^+^ lymphocyte populations significantly increased at 8 days of postinfection (Figure 3(a)). The increase of CD8^+^ cells was evident, but not significant (*p* < 0.05).

In order to estimate the proficiency of BM cells to internalize *B. abortus*, we performed an ex vivo infection. For this, BM cells were infected with *B. abortus*-RFP in the presence of anti-*Brucella* antibodies. As shown in Figure 3(b), close to 32% of the BM cells were infected; of these, over 90% were identified as PMNs [16].

Flow cytometry analysis of BM from infected mice rendered three main cell types containing *B. abortus*: monocytes, PMNs, and GMPs (Figure 3(c)). At 8 days of postinfection, the proportion of PMN-containing bacteria was greater than other cells. Strikingly, the number of infected PMNs dramatically decreased after 30 days. The proportion of infected monocytes remained similar at 8 and 30 days of postinfection. Although at early stages of infection close to 3% of the GMP-contained bacteria, the number of infected cells practically disappeared at later times (Figure 3(c)).

## 4. Discussion

At initial stages of infection, *Brucella* invades target organs, before a strong activation of the innate immune system and stimulation of antimicrobial mechanisms [7, 24]. This immunological gap allows the bacterium to colonize, replicate, and hide within cells of the mononuclear phagocyte system. Linked to this is the observation that *B. abortus* infection remains sequestered within BM cells for a protracted period, without significant changes in the bacterial loads. These phenomena propose a mechanism for *Brucella* persistence.

Granuloma formation, commonly observed in long-lasting infections, is an attempt to eliminate the microorganisms [25, 26]. In tuberculosis, it has been proposed that granulomas provide a bacterial safety shelter from the host immune response [27]. The higher number of granulomas in the BM and the permanence of these structures indicate the struggle of immune cells for eliminating *B. abortus*. This is also depicted by the significantly higher number of CD4^+^ lymphocytes in the BM at early stages of infection, which in brucellosis correlates with Th1 polarization [28].

The most abundant infected BM cells at the acute phase of murine infection (once antibodies against *Brucella* have developed) were PMNs. This result is reminiscent of the ex vivo infection of BM cells. Indeed, we have demonstrated that a large proportion of *ex vivo B. abortus*-infected BM cells are PMNs and that these leukocytes are unable to kill the ingested bacteria [16]. Despite this, it is unlikely that PMNs are the main reservoirs for *Brucella* in the BM. Indeed, *B. abortus* does not replicate in these cells and these infected leukocytes died prematurely [16]. Rather, PMNs may serve as vehicles for dispersing the bacterium, functioning as Trojan horses, as previously proposed [16, 18].

A small proportion of GMP cells in the BM were also infected at the acute phase of infection. This is unexpected since uncommitted progenitors such us GMP cells are not yet considered phagocytic cells [29]. However, at later time points, the proportion of infected cells was negligible. Moreover, the total number of GMP cells increased at early times of infection, diminishing afterward. These cellular variations correlate with the pathological changes of the BM. A similar phenomenon has been observed in human brucellosis cases [4, 30].

To our knowledge, this is the first time that myeloid oligopotent progenitor stem cells, lacking a developed phagocytic machinery, have been shown to become infected with *Brucella* organisms. Even though it is common to observe extramedullary hematopoiesis in the spleen of *Brucella*-infected mice [23], here we demonstrate for the first time *Brucella*-infected hematopoietic oligopotent stem cells residing in the BM. During emergency myelopoiesis, self-renewing GMPs in patches (pGMPs) build GMP clusters and differentiate into clustering GMPs (cGMPs). These GMP clusters can differentiate into mature cells until complete disappearance of the GMP clusters [31, 32]. Moreover, it has been shown that the increasing number of myeloid progenitors can promote microbial persistence in the organism [33]. All these findings make us speculate that *B. abortus* infects myeloid oligopotent progenitor stem cells and may interfere to induce GMP differentiation into infected-differentiated cells. Whether the reduced number of infected nonphagocytic erythrocytes and B cells [34, 35] originates from BM-infected progenitor cells remains unknown.

Despite the histopathological changes of the *B. abortus*-infected BM, and the low numbers of infected monocytes, the proportion of these leukocytes remained constant and persistent. It is well known that during granuloma formation, monocytes differentiate into macrophages, epithelioid cells, and dendritic Langerhans-type giant cells [36]. Moreover, *Brucella* is able to survive in monocytes and inhibits their programmed cell death [8]. Join-Lambert et al. [37] showed that *Listeria monocytogene*-infected myeloid cells in the bone marrow play a crucial role in the pathophysiology of meningoencephalitis by releasing infected cells into the circulation. Therefore, BM monocytes are firm candidates for *Brucella* reservoirs in the BM. These cells may be the source of the frequent relapses observed in antibiotic-treated individuals, even several years after the primo infection [38, 39].

## 5. Conclusions

Bacterial persistence, chronicity, and relapses are major problems in brucellosis. Within this context, we concluded (i) that loads of *B. abortus* in the BM remain constant and are long lasting; (ii) that *B. abortus*-infected BM displays histopathological modifications associated with augmented numbers of multipotent progenitor and active hematopoietic stem cells, PMNs, and CD4^+^ lymphocytes during the acute phase of the infection; and (iii) that the three types of infected cells in the BM are monocytes, PMNs, and GMP cells. In addition, we hypothesize that (iv) BM PMNs may serve as vehicles for dispersion of *Brucella*, following the Trojan horse hypothesis; (v) that *B. abortus*-infected myeloid oligopotent progenitor cells may differentiate into mature infected cells; and (vi) that monocytes are the most likely *Brucella* reservoirs in the BM and that these cells may be the source of the frequent relapses observed in antibiotic-treated individuals.

## Data Availability

The data used to support the findings of this study are included within the article.

## Supplementary Materials

Supplementary Materials Table S1: antibody staining scheme used for identifying each cell population.
