# Supplementary material for: Persistence of Brucella abortus in the Bone Marrow of Infected Mice
Source: J Immunol Res. 2018 Dec 3;2018:5370414. doi: 10.1155/2018/5370414 (PMC6304906; doi:10.1155/2018/5370414)
Supplement: Supplementary Materials — Table S1: antibody staining scheme used for identifying each cell population. [file 5370414.f1.pdf]

Table S1. Antibodies used for identifying each cell population

Cell type	Staining scheme
Granulocytes	$CD11b^{+}$ $Ly6G^{+}$
Neutrophils	$CD11b^{+}$ $CD115^{-}$ $Ly6G^{+}$
Eosinophils	$CD11b^{+}$ $Ly6G^{-}$ F4/80 SSC^{high}
GMP	$CD11b^{-}$ $CD3^{-}$ $Ly6G^{-}$ $CD19^{-}$ $cKit^{+}$ Sca^{low} $CD16/32^{+}$ $CD34^{+}$
MEP	$CD11b^{-}$ $CD3^{-}$ $Ly6G^{-}$ $CD19^{-}$ $cKit^{+}$ Sca^{low} $CD16/32^{-}$ $CD34^{-}$
Monocytes	$CD11b^{+}$ $CD115^{+}$ SSC^{low}
Macrophages (CD11b-)	$CD11b^{+}$ $Ly6G^{+}$ $F4/80^{+}$ SSC^{low}
Macrophages (CD11b+)	$CD11b^{-}$ $Ly6G^{-/low}$ $F4/80^{+}$ SSC^{low}
T cells (CD4+)	$CD11b^{-}$ $CD3^{+}$ $CD4^{+}$
T cells (CD8+)	$CD11b^{-}$ $CD3^{+}$ $CD4^{-}$ $CD8^{+}$
B cells	$B220^{+}$ $CD19^{+}$ $CD3^{-}$
